# Microscopic and Molecular Identification of *Sarcocystis* Species in Wild Brown Rats (*Rattus norvegicus*) in Lithuania and Latvia

**DOI:** 10.3390/ani16020331

**Published:** 2026-01-21

**Authors:** Giedrius Šidlauskas, Evelina Juozaitytė-Ngugu, Dalius Butkauskas, Petras Prakas

**Affiliations:** State Scientific Research Institute Nature Research Centre, Akademijos Str. 2, 08412 Vilnius, Lithuania; giedrius.sidlauskas@gamtc.lt (G.Š.); evelina.ngugu@gamtc.lt (E.J.-N.); dalius.butkauskas@gamtc.lt (D.B.)

**Keywords:** *Sarcocystis*, *Rattus norvegicus*, intestines, molecular identification, definitive host

## Abstract

*Sarcocystis* is a genus of heteroxenous, globally distributed apicomplexan parasites found in reptiles, birds, and mammals. Typically, sarcocysts develop in muscles of intermediate hosts, and oocysts sporulate in intestines of definitive hosts. The parasite’s life cycle is based on prey–predator relationships and usually involves two distinct host species. However, some *Sarcocystis* spp. can complete their full development within a single host species. Brown rats (*Rattus norvegicus*) are invasive, synanthropic, highly adaptable rodents that are true omnivores and opportunistic feeders. Therefore, it is possible that they can act as definitive hosts of *Sarcocystis* parasites. This study provides the first comprehensive assessment of *Sarcocystis* protists in brown rat intestinal samples under natural conditions, combining microscopy and molecular analyses. Of 27 brown rats investigated, 25.9% (7/27) of animals harbored oocysts/sporocysts of *Sarcocystis* spp. Based on nested PCR and sequencing of four genetic loci (*18S* rRNA, *28S* rRNA, ITS1, and *cox1*), 59.3% of samples were positive for *Sarcocystis* spp. Parasites identified were genetically similar to *Sarcocystis* spp. using bird–bird, bird–Carnivora, rodent–Carnivora, or ungulate–Carnivora as their intermediate–definitive hosts. The present study suggests that synanthropic rodents may facilitate cross-ecosystem transmission of these parasites, increasing infection pressure on livestock, companion animals, and wildlife in human-dominated environments. Future molecular and dietary ecological studies are needed to assess the role of synanthropic and opportunistic hosts, such as the brown rat, in the transmission of *Sarcocystis* spp.

## Simple Summary

The brown rat, an invasive and synanthropic rodent, exhibits highly opportunistic omnivorous feeding habits and plays an important role in the transmission of a wide range of pathogens to humans and domestic animals. *Sarcocystis* spp. are protists that alternate between two hosts, forming sarcocysts in muscle tissue of the intermediate hosts and developing sporocysts in the intestine of definitive hosts. Information on the role of brown rats as definitive hosts of *Sarcocystis* parasites under natural conditions is limited, with only *S. cymruensis* (syn. *S. rodentifelis*) having been experimentally confirmed to complete its life cycle in this rodent. Intestines of 27 brown rats were collected in Lithuania and Latvia and examined for *Sarcocystis* spp. The results of a light microscopy examination indicated that seven rats were positive for *Sarcocystis* spp. sporocysts and oocysts. Based on nested PCR and sequencing of several different genetic regions, more than half of the samples were positive for these parasites. Molecular analysis revealed the presence of DNA of several different *Sarcocystis* species that use rodents, ungulates, and birds as intermediate hosts. Overall, the study suggests that brown rats might be involved in the transmission of *Sarcocystis* species in nature.

## 1. Introduction

*Sarcocystis* parasites, which belong to the phylum Apicomplexa, are globally distributed protists that require two hosts to complete their life cycle [1]. The life cycle of *Sarcocystis* species is tightly linked to predator–prey interactions, with transmission occurring between prey animals serving as intermediate hosts (IH) and predators as definitive hosts (DH) [2,3]. *Sarcocystis* parasites occur in a wide range of hosts, from poikilothermic reptiles to homeothermic animals, namely mammals and birds. Sarcocyst development typically occurs in muscle tissue and the central nervous system (CNS) of IH. Meanwhile, oocysts sporulate in the small intestine of the DH [3]. To date, over 220 species of *Sarcocystis* have been documented across reptilian, avian, and mammalian hosts. Some *Sarcocystis* species are zoonotic [4]. Frequent infections of livestock by these parasites are associated with recurring annual losses in animal production [5]. Additionally, several *Sarcocystis* species are pathogenic to their IHs, including both wildlife and farm animals [1,2,6]. The severity of disease caused by *Sarcocystis* is determined by parasite species, infection load, location of sarcocyst formation, immune condition of the host, and other factors.

Various combinations of IHs and DHs in the life cycle of *Sarcocystis* have been identified [3]. Typically, *Sarcocystis* exhibits a diheteroxenous life cycle, in which carnivores, omnivores, or scavengers act as DHs and prey animals serve as IHs, meaning that different animal species are required to complete the parasite’s development [1]. The diversity of *Sarcocystis* species can vary significantly within the same IHs. For instance, raptors typically serve as IHs for only one or two *Sarcocystis* species [7,8]. In contrast, the mallard (*Anas platyrhynchos*) may harbor up to four species [9]. Even greater diversity has been observed in other hosts, with at least 13 *Sarcocystis* species identified in the red deer (*Cervus elaphus*) [10]. Certain *Sarcocystis* species that use mice, rats, and lizards as IHs (*S. cymruensis*, *S. dugesii*, *S. gallotiae*, *S. muris*, *S. simonyi*, and *S. stehlinii*) have been shown to exhibit dihomoxenous life cycles, meaning that they complete their entire development within a single host species [1]. These species can be transmitted through cannibalism or coprophagic behavior, either by consuming muscle tissue containing mature sarcocysts or ingesting feces from other individuals of the same host species [11,12,13,14,15,16,17].

Traditionally, research on *Sarcocystis* parasites has focused mainly on IHs, with a focus on extra-intestinal tissues, especially muscles [1,2,7,8,9]. Most research attention has focused on farm animals due to their economic importance and the impact of *Sarcocystis* infections on livestock production [18]. *Sarcocystis* was first described in Switzerland by Miescher in 1843, yet it took more than a century, until 1972, for transmission experiments to clarify its life cycle and identify the DHs of various *Sarcocystis* species [1]. In transmission experiments, different doses of sporocysts were experimentally administered to suspected IHs, and subsequent infections were monitored [13,14,15,16]. These foundational findings have significantly contributed to the current understanding of the sources of *Sarcocystis* infection, transmission dynamics, species identification and classification criteria, as well as key aspects of the parasite’s biology relevant to prevention and treatment strategies. However, the application of such transmission experiments is now significantly limited worldwide due to ethical regulations [19]. Experimental studies on *Sarcocystis* spp. under laboratory conditions are particularly rare for wild mammals and birds. Therefore, alternative approaches to elucidating the life cycles of these protists are essential. Advancements in molecular biology now allow the identification of *Sarcocystis* spp. through DNA analysis of fecal or intestinal samples from DHs [19,20,21,22,23,24]. The choice of molecular markers for *Sarcocystis* identification depends on the certain group of parasites. For example, the internal transcribed spacer 1 (ITS1) and the *28S* ribosomal RNA gene [20,21,22] are recommended for differentiating avian *Sarcocystis* species, while the mitochondrial cytochrome c oxidase subunit I (*cox1*) [19,21,23] is considered the most suitable marker for identifying *Sarcocystis* spp. that form sarcocysts in farmed animals and other ungulates.

Brown rats (*Rattus norvegicus*) serve as IHs for at least ten *Sarcocystis* species [1,24]. Among these, *S. singaporensis* and *S. cymruensis* are the most extensively studied [24,25,26,27,28,29,30,31,32]. The first species is important due to its pathogenicity and its potential use in rat control [26,27,28,30]. Notably, *S. cymruensis* forms macroscopic sarcocysts in the muscles of brown rats [24,25,29,32]. However, knowledge regarding the role of rats in transmitting these protists under natural conditions remains limited. Nevertheless, considering the dietary habits and ecological behavior of these invasive, synanthropic rodents, it is likely that they play a role in the natural transmission cycles of *Sarcocystis* spp. The brown rat is extremely adaptable, a true omnivore and highly opportunistic feeder [33,34]. They eat a broad spectrum of both plant and animal matter [35,36].

The brown rat inhabits a wide range of environments, especially those altered or associated with humans (e.g., urban areas, sewers, buildings, and refuse dumps) [33,34,35,36]. They also occur in rural landscapes (farmland, hedgerows, fields) and can survive in more natural settings when adequate shelter, food, and water are available [37]. As urban environments constitute a significant portion of the ecosystem encountered by human populations, pathogens carried by brown rats are potential candidates for spillover into humans [35,38]. For example, the brown rat can carry several zoonotic pathogens, including bacteria such as *Leptospira* [39], *Salmonella* [40], *Listeria* [41], and *Yersinia pestis* [42], as well as hantaviruses [43]. They are also capable of transmitting parasites like the tapeworm *Hymenolepis* [44] and protozoa such as *Giardia* [45].

The aim of this study was to assess the prevalence of *Sarcocystis* spp. in the intestines of brown rats using light microscopy and to identify the parasite species through PCR and sequencing.

## 2. Materials and Methods

### 2.1. Sample Collection

**Figure 1:**
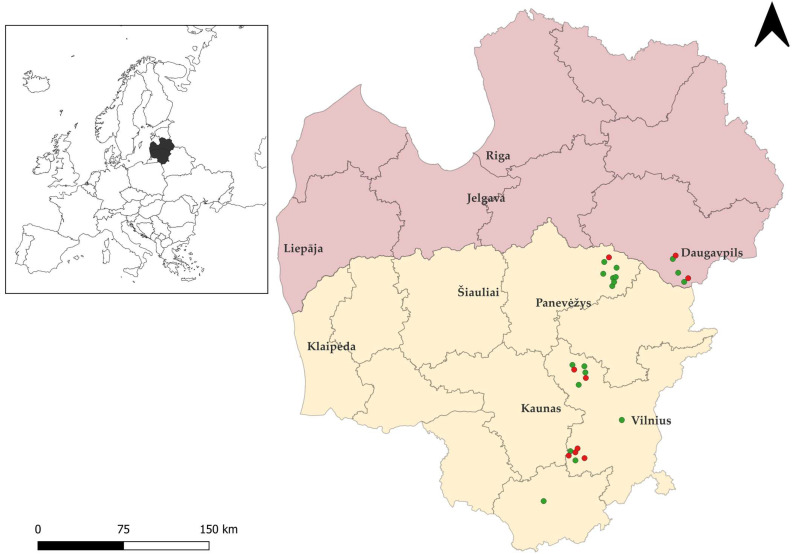
Geographic distribution of rodent sampling sites in Lithuania and Latvia created with Quantum Geographic Information System (QGIS, https://www.qgis.org, accessed on 11 December 2025). The territory of Lithuania is shown in yellow and that of Latvia in pink. Green circles indicate brown rat individuals positive for *Sarcocystis* spp., while red circles represent negative individuals.

Twenty-seven brown rats were snap-trapped between 2022 and 2025 in various regions of Lithuania and in the Daugavpils municipality of Latvia (Figure 1), were necropsied. Specifically, 22 rats were collected in Lithuania, and five were obtained from Latvia. Notably, in both countries studied, brown rats can be exterminated year-round without a permit [46]. Samples of brown rats were stored at −20 °C until microscopic examination. Intestinal samples were then examined for *Sarcocystis* spp., and oocysts/sporocysts were recovered from the intestinal mucosa of each animal using previously described methodology [47]. The resulting sediments were examined for oocysts/sporocysts under a Nikon ECLIPSE 8oi (Nikon Corp., Tokyo, Japan) light microscope (LM) at ×400 magnification. The 400 μL of re-suspended sediments were taken from each sample and used for DNA extraction. All samples underwent molecular analysis, regardless of whether oocysts/sporocysts were detected under LM.

### 2.2. DNA Extraction

DNA extraction from the intestinal scrapings of brown rats was conducted with the GeneJET Genomic DNA Purification Kit (Thermo Fisher Scientific Baltics, Vilnius, Lithuania) following the manufacturer’s recommendations. DNA samples were kept frozen at −20 °C until further analysis.

### 2.3. Molecular Identification of Sarcocystis Species

The role of brown rats in the natural transmission of *Sarcocystis* spp. within their environment is unknown. Therefore, a numerous set of primers targeting several different genetic regions, i.e., *18S* rRNA, *28S* rRNA, ITS1 and *cox1* were applied (Table 1). The specificity of primers in silico differed as some were specific to certain *Sarcocystis* species (*S. arvalis*, *S. cymruensis*, *S. muris*, *S. myodes* and *S. ratti* with rodents as their IHs), other primers were designed to amplify *Sarcocystis* spp. using certain groups of animals as their IHs and DHs, and lastly some primers used in the present work were genus specific. *Sarcocystis* species were amplified using the nested PCR (nPCR) approach. For *18S* rRNA, one primer pair was used in the first round of PCR, followed by two internal primer pairs in the second round. For *28S* rRNA, three primer pairs were used in the first round, and six pairs of internal primers were used in the second round, depending on the target species. For ITS1, a single primer pair was used in both the first and second rounds. For *cox1*, two primer pairs were used in the first round, and two internal primer pairs (one per target) were used in the second round.

The first round of nPCR was carried out in a 25 μL reaction containing 12.5 μL Taq Master Mix (Vazyme, Red Maple Hi-tech Industry Park, Nanjing, China), 7.5 μL nuclease-free water, 0.5 μL of each external primer, and 4 μL of extracted DNA. For the second amplification of nPCR, reactions were prepared in a 25 μL total volume, consisting of 12.5 μL Taq Master Mix (Vazyme, Red Maple Hi-tech Industry Park, Nanjing, China), 9.5 μL nuclease-free water, and 0.5 μL of each internal primer. However, instead of extracted genomic DNA, 2 μL of the first PCR product was used. Sterile water was used as the negative control in both nPCR rounds, while positive controls were included in all reactions except those for *S. muris*.

Both rounds of the nPCR were conducted according to the manufacturer’s protocol: beginning with the initial denaturation step at 95 °C for 3 min, followed by 35 cycles consisting of 15 s of denaturation at 95 °C, 15 s of annealing at 51–61 °C (depending on the primer pair used), and 60 s of elongation at 72 °C. A final extension was performed at 72 °C for 5 min. The efficiency of the reactions was verified using 1% agarose gel electrophoresis, and positive amplicons were enzymatically purified with ExoI and FastAP (Thermo Fisher Scientific Baltics, Vilnius, Lithuania) as per the manufacturer’s instructions.

All positive samples were subjected to direct sequencing using Big-Dye^®^ Terminator v3.1 Cycle Sequencing Kit (Thermo Fisher Scientific, Vilnius, Lithuania) and the 3500 Genetic Analyzer (Applied Biosystems, Foster City, CA, USA). Both forward and reverse primers of the second round of nPCR were employed for Sanger sequencing. Obtained sequences were compared to most closely related *Sarcocystis* spp. using Nucleotide BLAST (https://blast.ncbi.nlm.nih.gov/, accessed on 10 November 2025). Phylogenetic analyses were performed in MEGA7.0.26 [54] using the Maximum Likelihood method. Bootstrap support was calculated based on 1000 replicates, and to enhance readability, values < 50% were omitted from the final phylogenetic trees. The sequences generated in the present study are available in GenBank with accession numbers PX550574–PX550577 (ITS1), PX571985–PX571989 (*28S* rRNA), PX571990–PX571991 (*18S* rRNA), PX577581–PX577584 (*cox1*).

## 3. Results

### 3.1. Microscopic Identification of Sarcocystis spp. in Intestine Samples

**Figure 2:**
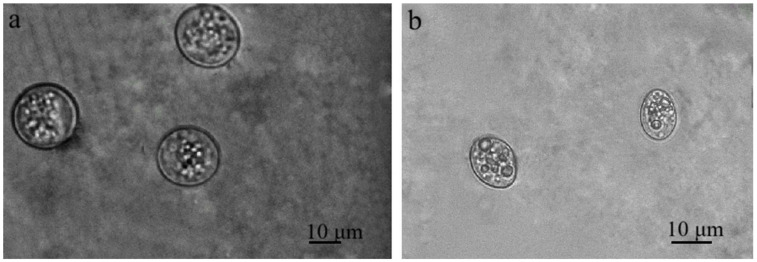
*Sarcocystis* spp. Sporulating oocysts (**a**) and sporocysts (**b**) in the intestinal epithelium of small intestines of brown rat.

*Sarcocystis* spp. oocysts and/or sporocysts were observed in the intestinal epithelium under the LM (Figure 2). The number of oocysts and/or sporocysts detected in the area of the 24 × 24 mm coverslip ranged from one to five, with oocysts being more frequently observed than sporocysts. The prevalence of *Sarcocystis* spp. in mucosal scrapings of brown rats was 25.9% (7/27). Under LM, free sporocysts measuring 8.8–14.3 × 10.0–18.8 μm (10.9 ± 2.6 × 13.7 ± 1.7 μm, *n* = 24) were observed (Figure 2a). Oocysts of *Sarcocystis* spp. 14.0–19.5 × 15.4–23.0 μm (16.1 ± 1.7 × 19.3 ± 2.8 μm, *n* = 18) were also noticed (Figure 2b). It is noteworthy that oocysts and sporocysts were observed in one intestinal epithelial sample from a brown rat (RnLt25z), while in other samples, either sporocysts or oocysts were detected.

### 3.2. Molecular Characterization of Sarcocystis spp. in Brown Rats

Having examined the intestines of brown rats, we obtained 15 pure DNA sequences of *Sarcocystis* spp. (Table 2). Fragments of four different genetic loci, *18S* rRNA, *28S* rRNA, ITS1, and *cox1*, were amplified using seven different pairs of primers. BLAST analysis indicated that the sequences obtained in this study corresponded to six previously described *Sarcocystis* species, whereas four sequences could not be reliably assigned to species level and were therefore classified as *Sarcocystis* sp. In the case of *S. alces*, *S. capreolicanis*, and *S. gracilis*, our sequences showed 98.8–100% similarity to those of certain identified species and displayed ≤95.3% similarity to those of other *Sarcocystis* species. Beyond that, our 313 bp *28S* rRNA sequence amplified with SgrauzinF/SspRod1R demonstrated 100% identity to *S. arvalis* and showed ≤97.8% similarity to those of other species. Additionally, four 282 bp ITS1 sequences generated with the primers HSpauk3/HSpauk4 displayed 96.8–99.7% similarity between themselves and showed up to 99.3–100% similarity (specifically, 97.2–100%, 95.4–99.3%, 96.8–99.7%, and 96.5–99.3% similarity) to those of *S. halieti*. These four sequences showed similarity of 94.7–97.2% to that of *Sarcocystis* sp. isolate Skua-2016-CH (MW160469). Furthermore, the 337 bp *cox1* sequence amplified using SsunV1b/SsunV2b exhibited the highest values of similarity to those of *S. tenella* (98.8–100%) and *S. capracanis* (97.0–98.5%) (Table 2).

Three sequences that could not be assigned to certain *Sarcocystis* species showed very high and comparable sequence similarity values to several *Sarcocystis* spp. with a rodent (*Sarcocystis* sp. Ziu1) or bird (*Sarcocystis* sp. Ziu2 and *Sarcocystis* sp. Ziu3) as their IHs. Finally, the 713 bp *28S* rRNA sequence of *Sarcocystis* sp. Ziu4 displayed ≤99.0% similarity to those of other *Sarcocystis* spp. and demonstrated the highest values of similarity to species forming sarcocysts in muscles of birds (*S. wenzeli*, *S. cristata*, *S. albifronsi*, and *S. anasi*).

Additionally, we have obtained seven sequences that showed double peaks. These sequences were detected in seven separate brown rats (Table 2), demonstrated at least 84.9% similarity with *Sarcocystis* spp., and had query coverage values of from 72% to 100%. Two ITS1 sequences obtained using HSpauk3/HSpauk4 displayed the highest similarity to those of *S. columbae* and *S. halieti* parasitizing birds. A single impure *28S* rRNA sequence exhibited the highest similarity to those of *S. alces* with moose (*Alces alces*) as IH. Finally, four *cox1* sequences showed the highest similarity to those of *Sarcocystis* spp. (*S. gracilis*, *S. linearis*, and *S. tenella*) from domestic and wild ungulates. Out of 27 examined brown rats, oocysts and/or sporocysts were morphologically detected in seven (25.9%) samples and molecularly in 16 (59.3%) samples. Two samples were positive only by morphological examination but yielded no *Sarcocystis* DNA, whereas 11 samples were positive only by molecular analysis. Five samples were positive by both methods, and nine samples were negative by both morphological and molecular examinations. When only samples from animals yielding pure DNA sequences were considered, the molecular detection rate of *Sarcocystis* spp. was 44.4% (12/27).

**Figure 3:**
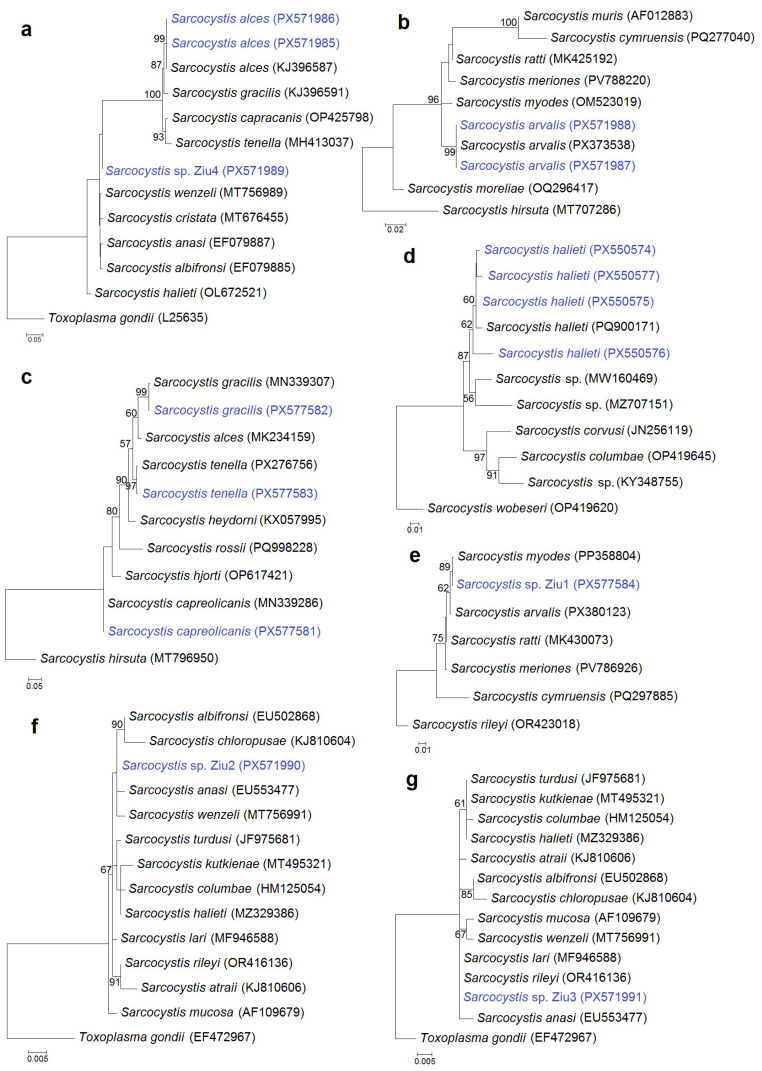
Phylogenetic trees of selected *Sarcocystis* species based on *28S* rRNA (**a**,**b**), *cox1* (**c**,**e**), ITS1 (**d**), and *18S* rRNA (**f**,**g**) gene sequences. The trees were constructed using the maximum likelihood method and rooted with *Toxoplasma gondii* (**a**,**f**,**g**), *S. hirsuta* (**b**,**c**), *S. wobeseri* (**d**), and *S. rileyi* (**e**). The following nucleotide substitution models were applied: T92 + G (**a**,**e**), HKY + G (**b**), K2 + G (**c**), K2 (**d**,**g**), and T92 (**f**). Bootstrap values are shown next to the branches. *Sarcocystis* spp. obtained in this study are shown in blue.

Phylogenetic analyses were performed to confirm species identification and to resolve phylogenetic relationships of the detected *Sarcocystis* spp. As primer sets targeting four genetic markers and different regions within the same gene were analyzed, seven separate phylogenetic trees were constructed using *28S* rRNA (Figure 3a,b), *cox1* (Figure 3c), ITS1 (Figure 3d), and *18S* rRNA (Figure 3e,f,g) sequence datasets. Sequences of *S. alces*, *S. arvalis*, *S. gracilis*, *S. tenella*, *S. capreolicanis*, and *S. halieti* grouped with reference sequences of their respective species, supporting their molecular identification. In contrast, the variability of some genetic regions analyzed was not sufficient to reliably resolve four *Sarcocystis* spp., which clustered among several closely related *Sarcocystis* species. *Sarcocystis* sp. Ziu1 was grouped with species that use rodents as IHs, forming a clade with *S. myodes*, *S. arvalis*, *S. ratti*, and *S. meriones*, with a closer relationship to *S. myodes*. By comparison, *Sarcocystis* sp. Ziu2, *Sarcocystis* sp. Ziu3, and *Sarcocystis* sp. Ziu4 were placed with species that use birds as IHs and carnivores as DHs. Notably, *Sarcocystis* sp. Ziu4 differed by ≥1% at the conserved *28S* rRNA locus from phylogenetically related taxa and formed separate branch in the phylogram (Figure 3a), indicating a genetically distinct, previously uncharacterized *Sarcocystis* taxon.

In two brown rats (RnLt24z and RnLt25z), *S. arvalis*, which uses rodents and carnivorous mammals as hosts [55], was identified. In addition, a sequence showing the highest similarity to *Sarcocystis* species using ungulates as IHs was detected in the same animal, indicating that DNA of two different *Sarcocystis* species was most likely present in these isolates. Likewise, *S. alces*, which employs moose as its IH, was identified in the brown rat RnLt21z. In this individual, an additional sequence with double peaks and the highest similarity to *Sarcocystis* spp. forming sarcocysts in bird muscles was also obtained, indicating a mixed infection with at least two *Sarcocystis* species. Furthermore, two distinct *Sarcocystis* species were certainly found in the brown rat RnLt14z, while three separate species were established in the rat RnLv4z. Based on 15 unambiguous sequences obtained, the detected *Sarcocystis* species were characterized by the following IH–DH associations: bird–bird (4 cases), bird–Carnivora (3), rodent–Carnivora (3), and ungulate–Carnivora (5). In five positive brown rats by both morphological and molecular analyses, *S. arvalis*, *S. halieti*, and *Sarcocystis* sp. Ziu1, which demonstrated the highest genetic similarity to *Sarcocystis* with rodents as IH, were identified.

## 4. Discussion

To our knowledge, this study provides preliminary evidence of the presence of *Sarcocystis* spp. in the intestinal epithelium of brown rats under natural conditions. *Sarcocystis* spp. oocysts/sporocysts were detected by LM in 25.9% (7/27) of brown rats examined. In comparison, the presence of *Sarcocystis* spp. DNA was confirmed by molecular analysis in 16 (59.3%) mucosal scrapings from brown rats. Comparative analysis of the obtained sequences revealed the highest similarity to species that employ birds, rodents, and ungulates as IHs. According to previous experimental studies, oocysts/sporocysts of *S. cymruensis* were detected in mucosal scrapings and within the remaining wall of the small intestine in 23 (88.5%) Wistar (Norway) laboratory rats [15]. To date, no additional data are available regarding brown rats as potential DHs for *Sarcocystis* spp.

Evolutionarily, parasites that have only one DH persist by using several IH species in their life cycle [56,57]. Consequently, parasite life cycles restricted to one IH and one DH species represent an evolutionary paradox, particularly when infection can occur in only one host through ingestion of sporocysts. Numerous experimental transmission attempts of *Sarcocystis* spp. from diverse DHs to brown rats as IHs have been unsuccessful [58,59,60], apart from the case of *S. singaporensis*, in which successful transmission was achieved [27]. While transmission experiments are still regarded as the gold standard for identifying the DHs of *Sarcocystis* species, their use has become increasingly limited due to high costs, lengthy duration, and significant ethical considerations [61]. In this study, *Sarcocystis* spp. were confirmed by both microscopical and molecular analyses in five brown rats. In the intestines of two brown rats (RnLt11z and RnLt12z), oocysts or sporocysts of *Sarcocystis* spp. were detected by light microscopy; however, no DNA sequences were obtained using the primer set applied in this study. Furthermore, molecular screening alone detected *Sarcocystis* DNA in the intestines of 11 additional brown rats. It is not possible to rule out pseudoparasitism (the organism is found in a host but is not actually acting as a parasite) for certain *Sarcocystis* species detected in the intestinal epithelium of brown rats. Additionally, the detection of *Sarcocystis* DNA in intestinal samples does not unequivocally demonstrate endogenous sporocyst formation, since the DNA may originate from species ingested with the rodents’ diet [62]. In this study, sporocysts characteristic of *Sarcocystis* were identified in three intestinal samples of brown rats (RnLt11z, RnLt21z, and RnLt25z), suggesting that these rodents may act as DHs. Additionally, oocysts were detected in the intestinal epithelium of several individuals (RnLv5z, RnLt10z, RnLt12z, RnLt15z, and RnLt25z).

Considering the ecological traits and feeding habits of invasive, synanthropic brown rats [35,36,63,64], their involvement in the natural transmission cycles of *Sarcocystis* spp. cannot be excluded. The brown rat represents one of the most successful commensal pests in human history, profoundly influencing numerous aspects of human life [65]. Brown rats are known to exhibit muricidal behavior, wherein they kill and devour mice [66,67]. This behavior serves dual functions, reducing interspecific competition and exploiting mice as a convenient food resource. Furthermore, brown rats display pronounced scavenging tendencies and readily consume degraded animal tissues, including fish, avian, and mammalian remains, most often sourced from human-generated waste or natural carrion [68,69].

In the present study, six *Sarcocystis* spp. (*S. alces*, *S. arvalis*, *S. capreolicanis*, *S. gracilis*, S. *halieti*, and *S. tenella*) were identified in the intestinal epithelium of brown rats for the first time. It is noteworthy that canids act as the DHs for *S. alces* [23,70], *S. capreolicanis* [47,71,72], *S. gracilis* [71,72], and *S. tenella* [23,72]. Meanwhile, molecular analyses indicated that three sequences of unidentified *Sarcocystis* species (*Sarcocystis* sp. Ziu2, *Sarcocystis* sp. Ziu3, and *Sarcocystis* sp. Ziu4) were most genetically similar to bird muscle–infecting species (*S. albifronsi*, *S. anasi*, *S. atraii*, *S. cristata*, *S. rileyi*, and *S. wenzeli*) transmitted by carnivores [61]. In this work, *S. halieti* was one of the most commonly detected species (n = 4). Molecular analyses indicate that the DHs of *S. halieti* are raptors belonging to the orders Accipitriformes [20,62,73,74,75] and Falconiformes [62]. Therefore, it is unlikely that brown rats serve as DHs for the above-mentioned *Sarcocystis* spp. Instead, these *Sarcocystis* species were probably associated with carrion consumption and failed to establish infection within the host.

Based on the comparison of the obtained *28S* rRNA sequences, for the first time, *S. arvalis* has been identified in the intestinal samples of two brown rats. Recently, *S. arvalis* was described in the muscles of the common vole (*Microtus arvalis*) from Lithuania [55]. The DH of this species remains undetermined; however, based on phylogenetic relationships, predatory mammals are considered the most likely hosts. Alternative transmission routes for *S. arvalis* cannot be excluded, including cannibalism or coprophagy, whereby individuals consume muscle tissue harboring mature sarcocysts or feces from conspecific hosts. A similar scenario may pertain to the putative new *Sarcocystis* sp. Ziu1, with rodents as IHs.

Our study suggests that brown rats might be involved in the transmission of *Sarcocystis* species in nature. Sporocysts of *Sarcocystis* spp. were detected in three intestinal scrapings of brown rats, indicating that the sexual stage of the parasite may be completed in these rodents. However, the detected numbers of sporocysts and/or oocysts were low when compared with those typically found in DHs such as canids [23,47], felids [1], wild birds [22,75], and snakes [1,76]. Taken together, these results suggest that brown rats are unlikely to represent typical DHs. However, under certain ecological conditions, such as occasional coprophagy or cannibalism, they may potentially act as DHs, with sporocyst excretion occurring at low intensity but possibly over an extended period [15]. These findings suggest that brown rats may play a previously underappreciated role in maintaining *Sarcocystis* transmission cycles in human-dominated landscapes, potentially affecting domestic animals and wildlife. Future studies integrating large-scale molecular screening, phylogenetic analyses, dietary ecology, and histological examination of intestinal tissues will be essential to identify DHs of *Sarcocystis* species under natural conditions, particularly in synanthropic and opportunistic hosts such as the brown rat.

## 5. Conclusions

This study provides the first detailed examination of intestinal samples from brown rats for *Sarcocystis* protists under natural conditions using combined microscopical and molecular approaches. Molecular screening showed higher sensitivity than light microscopy (59.3% vs. 25.9%). Based on nPCR and sequencing of *18S* rRNA, *28S* rRNA, ITS1, and *cox1* fragments, six previously described *Sarcocystis* species and four genetically distinct parasite lineages were detected. The identified *Sarcocystis* spp. were associated with bird–bird, bird–Carnivora, rodent–Carnivora, and ungulate–Carnivora IH–DH relationships, indicating that in most cases brown rats do not serve as true DHs. Nevertheless, the detection of low numbers of sporocysts and oocysts in several individuals suggests that brown rats may occasionally complete the sexual life cycle of certain *Sarcocystis* species, possibly through coprophagy or cannibalism. Overall, brown rats are unlikely to represent typical DHs but may play a limited, context-dependent role in *Sarcocystis* transmission in nature.

## Figures and Tables

**Table 1:** The oligonucleotides used in this study and their characteristics.

Locus	Primer	Primer Sequence	Target Species	F/R *	Round of Nested PCR	Product Length, bp	Ref.
*18S* rRNA	SUNIF1	ACCTGGTTGATCCTGCCAGT	*Sarcocystis* spp.	F	I	~1000	[24]
*18S* rRNA	SUNIR1	TTCGCAGTAGTTCGTCTTTAACA	*Sarcocystis* spp.	R	I	~1000	[24]
*18S* rRNA	SarAF	CTGGTTGATCCTGCCAGTAG	*Sarcocystis* spp.	F	II	525	[48]
*18S* rRNA	SarAR	TTCCCATCATTCCAATCACT	*Sarcocystis* spp.	R	II	525	[48]
*18S* rRNA	SarBF	GGGAGGTAGTGACAAGAAATAACAA	*Sarcocystis* spp.	F	II	500	[48]
*18S* rRNA	SarBR	GGCAAATGCTTTCGCAGTAG	*Sarcocystis* spp.	R	II	500	[48]
*28S* rRNA	Sgrau281	GAACAGGGAAGAGCTCAAAGTG	*Sarcocystis* spp.	F	I	898	[49]
*28S* rRNA	Sgrau282	GGTTTCCCCTGACTTCATTCTAC	*Sarcocystis* spp.	R	I	898	[49]
*28S* rRNA	Sgrau283	GAGAGGCGTTACCAGTGGAG	*Sarcocystis* spp.	F	II	780	[49]
*28S* rRNA	Sgrau284	CAGATATGCTCCAACTCAAACCTCT	*Sarcocystis* spp.	R	II	780	[49]
*28S* rRNA	SgrauzinF	CCTGTGTCATTTAGTTCCACGTA	*S. myodes*	F	II	420	PS
*28S* rRNA	SmyodesR	TAAAAAGAAAAGTTCCAACGGTGT	*S. myodes*	R	II	420	PS
*28S* rRNA	SgrauzinF	CCTGTGTCATTTAGTTCCACGTA	*S. ratti*	F	II	357	PS
*28S* rRNA	SrattiR	CCAGAATCCTTTCACCCCAAC	*S. ratti*	R	II	357	PS
*28S* rRNA	SgrauzinF	CCTGTGTCATTTAGTTCCACGTA	*S. arvalis*	F	II	357	PS
*28S* rRNA	SspRod1R	CTGGAGTCTTTTCGTCCCAAC	*S. arvalis*	R	II	357	PS
*28S* rRNA	28Mur1	TGGGAATCGGAGATTTTTGA	*S. muris*	F	I	492	PS
*28S* rRNA	28Mur2	TACCTGCTACACTGGGGGTCT	*S. muris*	R	I	492	PS
*28S* rRNA	28Cymmur3	CTGTGTCGTTTAGGTCCCACGGA	*S. muris*/ *S. cymruensis*	F	II	322	PS
*28S* rRNA	28Mur4	CCAATACGATCCTCACCCACT	*S. muris*	R	II	322	PS
*28S* rRNA	28Cym1	GGGAATCGAAGATTTTTGAGTGG	*S. cymruensis*	F	I	600	PS
*28S* rRNA	28Cym2	GTACTCGAGTATTGCTACTCAAG	*S. cymruensis*	R	I	600	PS
*28S* rRNA	28Cymmur3	CTGTGTCGTTTAGGTCCCACGGA	*S. muris*/ *S. cymruensis*	F	II	438	PS
*28S* rRNA	28Cym4	TGCTAATAGTACACTTCAGTCGTGA	*S. cymruensis*	R	II	438	PS
ITS1	HSpauk1	ATCATTGCCTATGTATGTCATGTATAT	IH = birds	F	I	516	[50]
ITS1	HSpauk2	CAGAGTCCAAGGCGATAGAAAT	IH = birds	R	I	516	[50]
ITS1	HSpauk3	TAAGGGAATTTGTGGTTGGA	IH = birds	F	II	327	[50]
ITS1	HSpauk4	GATAAAAGAATACGATGTGAGAAAAA	IH = birds	R	II	327	[50]
*Cox1*	SF1	ATGGCGTACAACAATCATAAAGAA	*Sarcocystis* spp.	F	I	~1000	[51]
*Cox1*	SelniaiR	AAATAYCTTRGTGCCCGTAG	IH = ungulate	R	I	~1000	[52]
*Cox1*	SkiaunbvV1	GCCCAGAATTAACGCCATT	IH = ungulate	F	II	429	PS
*Cox1*	SkiaunbvV2	AACCAAAAYAAGTGCTGRTACAGT	IH = ungulate	R	II	429	PS
*Cox1*	SF1	ATGGCGTACAACAATCATAAAGAA	*Sarcocystis* spp.	F	I	913	[51]
*Cox1*	SsunR3	CCGTTGGWATGGCRATCAT	IH = ungulate, DH = Canidae	R	I	913	[53]
*Cox1*	SsunV1b	CAGGTATCTTTAGYGTTGTTGG	IH = ungulate, DH = Canidae	F	II	378	PS
*Cox1*	SsunV2b	AGTCAACGGCCTCCGTATT	IH = ungulate, DH = Canidae	R	II	378	PS
*Cox1*	SF1	ATGGCGTACAACAATCATAAAGAA	*Sarcocystis* spp.	F	I	~1100	[51]
*Cox1*	SR5	TAGGTATCATGTAACGCAATATCCAT	*Sarcocystis* spp.	R	I	~1100	[51]
*Cox1*	SgraucoF	GGTTTTGGTAACTACTTTGTACCG	IH = rodent	F	II	660	[49]
*Cox1*	SgraucoR	ACCTCTAATCCTACGGTCATCA	IH = rodent	R	II	660	[49]
*Cox1*	SF1	ATGGCGTACAACAATCATAAAGAA	*Sarcocystis* spp.	F	I	~1000	[51]
*Cox1*	SkatR2	GCTGAACAGTATTACGAATGATATG	IH = ungulate, DH = Canidae	R	I	~1000	PS
*Cox1*	SkatV1	AGTTTGGCGCTGCCGTAG	IH = ungulate, DH = Felidae	F	II	370	PS
*Cox1*	SkatV2	TCAGGGTGCCCGAAGAAC	IH = ungulate, DH = Felidae	R	II	370	PS

IH—intermediate host, DH—definitive host, *—orientation of primers, forward (F) or reverse (R), Ref—reference, PS—present study.

**Table 2:** Genetic identification of *Sarcocystis* species in intestines of brown rats from Lithuania and Latvia.

Isolate	Microscopic Examination *	Gene	Sequence	Genetic Similarity, %	Species	IH	DH
RnLt1z	Negative	ITS1	Pure	97.9–100% *S. halieti*, 96.8% *Sarcocystis* sp. (MW160469), 94.5% *Sarcocystis* sp. (MZ707151)	*S. halieti*	Birds	Birds
RnLv4z	Negative	*18S* rRNA	Pure	100% *S. rileyi*, 99.8% *S. atraii*, 99.6% *S. albifronsi*	*Sarcocystis* sp. Ziu3	Birds	Carnivora
		*28S* rRNA	Pure	98.7–99.0% *S. wenzeli*, 98.2% *S. cristata*, 98.0% *S. albifronsi*	*Sarcocystis* sp. Ziu4	Birds	Carnivora
		*cox1*	Pure	98.8–100% *S. tenella*, 97.0–98.5% *S. capracanis*, 95.3–95.9% *S. alces*	*S. tenella*	Sheep	Carnivora
RnLv5z	1 oocyst	*18S* rRNA	Pure	99.8 –100% *S. myodes*, 99.7% *S. arvalis*, 99.2% *S. ratti*	*Sarcocystis* sp. Ziu1	Rodents	Carnivora
RnLv6z	Negative	*18S* rRNA	Pure	99.8% *S. albifronsi* and *S. anasi*, 99.7% *S. rileyi*	*Sarcocystis* sp. Ziu2	Birds	Carnivora
RnLt8z	Negative	ITS1	DP	84.9% *S. linearis* (QC = 88)	–	–	–
RnLt9z	Negative	*cox1*	DP	87.9% *S. halieti* (QC = 95)	–	–	–
RnLt10z	1 oocyst	ITS1	Pure	98.2–100% *S. halieti*, 97.2% *Sarcocystis* sp. (MW160469), 94.7% *Sarcocystis* sp. (MZ707151)	*S. halieti*	Birds	Birds
RnLt11z	2 sporocysts	–	–	–	–	–	–
RnLt12z	5 oocysts	–	–	–	–	–	–
RnLt14z	Negative	*cox1*	Pure	99.4–100% *S. capreolicanis*, 94.7–95.0% *S. hjorti* and *S. pilosa*	*S. capreolicanis*	Roe deer	Carnivora
		ITS1	Pure	97.5–100% *S. halieti*, 94.7% *Sarcocystis* sp. (MW160469), 92.9% *Sarcocystis* sp. (MZ707151)	*S. halieti*	Birds	Birds
RnLt15z	3 oocysts	ITS1	Pure	98.9–100% *S. halieti*, 96.5% *Sarcocystis* sp. (MW160469), 94.0% *Sarcocystis* sp. (MZ707151)	*S. halieti*	Birds	Birds
RnLt16z	Negative	*28S* rRNA	Pure	99.6–100% *S. alces*, 92.8–94.1% *S. capracanis*, 92.3–93.4% *S. tenella*	*S. alces*	Moose	Carnivora
RnLt17z	Negative	*cox1*	Pure	98.8–100% *S. gracilis*, 95.0–95.3% *S. alces*, 93.8–94.4% *S. tenella*	*S. gracilis*	Roe deer	Carnivora
RnLt21z	5 sporocysts	*28S* rRNA	Pure	99.6–100% *S. alces*, 92.8–94.1% *S. capracanis*, 92.3–93.4% *S. tenella*	*S. alces*	Moose	Carnivora
		ITS1	DP	89.9% *S. columbae* (QC = 72)	–	–	–
RnLt22z	Negative	*cox1*	DP	97.6% *S. gracilis* (QC = 99)	–	–	–
RnLt24z	Negative	*28S* rRNA	Pure	100% *S. arvalis*, 96.8–97.8% *S. myodes*, 97.8 *S. ratti*	*S. arvalis*	Rodentia	Carnivora
		*cox1*	DP	93.2% *S. gracilis* (QC = 99)	–	–	–
RnLt25z	3 sporocysts, 1 oocyst	*28S* rRNA	Pure	100% *S. arvalis*, 96.8–97.8% *S. myodes*, 97.8 *S. ratti*	*S. arvalis*	Rodentia	Carnivora
		*28S* rRNA	DP	93.2% *S. alces* (QC = 81)	–	–	–
RnLt26z	Negative	*cox1*	DP	94.9% *S. tenella* (QC = 100)	–	–	–

DP sequences with double peaks, *Sacocystis* species could not be defined, * Number of sporocysts/oocysts of *Sarcocystis* spp. in 20 µL of suspension in intestinal scrapings, IH—intermediate host, DH—definitive host. Notably, RnLv4z, RnLv5z and RnLv6z isolates were collected in Latvia, while other isolated originated from Lithuania.

## Data Availability

The sequences generated in the present study have been deposited in GenBank under the accession numbers PX550574–PX550577 (ITS1), PX571985–PX571989 (*28S* rRNA), PX571990–PX571991 (*18S* rRNA), and PX577581–PX577584 (*cox1*).
